# Improving performance of mesoporous MOF AlTp impregnated with ionic liquids for CO_2_ adsorption

**DOI:** 10.1038/s41598-023-30267-x

**Published:** 2023-02-24

**Authors:** Narmin Noorani, Abbas Mehrdad

**Affiliations:** Department of Physical Chemistry, Faculty of Chemistry, University of Tabriz, Tabriz, Iran

**Keywords:** Energy science and technology, Carbon capture and storage

## Abstract

In this work, the CO_2_ adsorption performance of metal–organic frameworks (MOFs) impregnated with ionic liquids (ILs) was studied using quartz crystal microbalance (QCM) at the temperature of 298.15 K and pressures up to 5 bar. The hybrid composites consist of aluminum terephthalate metal–organic framework (AlTp) impregnated of 1-butyl-4-methyl pyridinium and 1-butyl-3-methylimidazolium–based ionic liquids (ILs) with different anions, viz. tetrafluoroborate ([BF_4_]^−^), thiocyanate ([SCN]^−^), chloride ([Cl]^−^), and bromide ([Br]^−^). ILs-impregnated AlTp synthesized was characterized using scanning electron microscopy (SEM), X-ray diffraction analysis (XRD), the thermogravimetry analysis (TGA) and Fourier transform infrared (FTIR) spectroscopy. CO_2_ adsorption isotherms of the IL/AlTp composites and AlTp were measured to evaluate the ILs effect on the CO_2_ adsorption of the AlTp. Comparison of CO_2_ adsorption in ILs/AlTp with different anion ([Cl]^−^, [Br]^−^, [SCN]^−^, [BF_4_]^−^) reveals that CO_2_ adsorption in ILs/AlTp was increased in the order as: [BF_4_]^−^ < [SCN]^−^ < [Br]^−^ < [Cl]^−^. The results show that [BMPyr][Cl]/AlTp the highest CO_2_ adsorption capacity, 2.6 times higher than that of AlTp at 5 bar and 298.15 K which helps to guide the logical design of new mixtures for gas separation applications. Also, adsorption/desorption test show that regeneration performance of [BMPyr][Cl]/AlTp is 96.53% after five consecutive cycles adsorption/desorption.

## Introduction

Climate change is one of the most significant challenges ago for countries around the world; preventing and modifying it received widespread attention^1^. Rising global temperatures pose an urgent threat to the planet, with an increase of approximately 2 °C by the end of this century^2^. The increase in the average temperature of the earth can be attributed to a group of greenhouse gases, of which carbon dioxide (CO_2_) accounts for more than 70% of the total^3,4^. To overcome these troubles, the capture potential and storage concept (CCS) for CO_2_ was offered to control the CO_2_ amount in the atmosphere^5^. Different CO_2_ capture technologies are presently accessible containing absorption (physical and chemical absorptions), cryogenic separation, adsorption, and membrane technologies. Among these technologies, the chemical absorption of amine scrubbing is used as an efficient technique for the chemical adsorption of CO_2_ in industries^6,7^. Although the usage of the amine method can reduce CO_2_ by up to 98%, this technique is inefficient with a large number of absorber requirements, high energy consumption, corrosions, etc. As a result, solid adsorption processes have been studied to reduce these problems^8^. A significant category of porous materials has been investigated as adsorbents, such as activated carbon, zeolites, aluminophosphates, silica gel, carbon nanotubes, polymeric resins, and porous metal–organic frameworks^9,10^.

Among the mentioned adsorbents, porous metal–organic frameworks (MOFs) have received the attention of many researchers owing to their high adsorption capacity and significant selectivity for the past two decades. Metal–organic frameworks (MOFs) are a category of porous materials, which their structure is composed of metal ions networks or metal ion clusters and organic linkers connected through coordination bonds^11^. MOFs own a high internal surface area, tunable multifunctional pores, adaptable porosity, and high thermal and chemical stabilities^12,13^. MOF structure and properties can be tuned using selecting the appropriate linker metal pair for a purpose application^14^, such as the adsorption of specific gas species^15,16^. Improving the pore characteristics of MOFs with different active sites, among which creation of incorporation of carboxylate oxygen atoms/–NH_2_ or uncoordinated nitrogen/ and or adjustment of pore space partition, and unsaturated metal centers (UMCs), can increase preferential interactions between the frameworks and CO_2_^17,18^. Recently, MOFs have been modified using functional molecules impregnation and chemical functionalization to further enhance their CO_2_ uptake performance. Because of the complex manipulation of chemical function, physical modification is a convenient alternative by introducing functional molecules in metal–organic frameworks that show a lot of tendency for CO_2_. Recent investigations have focused on improving the CO_2_ adsorption performance of MOFs using the impregnation of ionic liquids (ILs) on the porous frameworks.

Ionic liquids are molten solvent having melting point below 373 K, and considered as green solvents, have potential as a possible replacement candidate for conventional solvents especially in the area of green chemistry and have been described as potential environmentally in a variety of applications^19,20^. Their unique properties include low volatility, high thermal stability, and recyclability^21^. The possibility to design their physicochemical properties by tuning the anion or cation in their structure makes them unique compared to other organic solvents^22–24^. Azevedo et al.^25^ studied the adsorption of pure methane, carbon dioxide, nitrogen, and typical mixtures found in natural gas and flue gases in mesoporous MOF MIL-100(Fe) impregnated with ILs [Bmim][Tf_2_N] and [Bmim][PF_6_] using molecular simulations. Vicent-Luna et al. ^26^ analyzed the adsorption and diffusion of gases such as CO_2_, N_2_, and CH_4_ in metal–organic frameworks (MOFs) consisting of IRMOF-1, HMOF-1, MIL-47, and MOF-1 impregnated of 1-ethyl-3-methylimidazolium thiocyanate. They have suggested that the increase the gas adsorption and controlling the pore sizes of the structures to further selective adsorption. Gupta et al. ^27,28^ investigated CO_2_ capture in IRMOF-1 supported four IL including cation 1-*n*-butyl-3-methylimidazolium [BMIM]^+^ with various anions: tetrafluoroborate [BF_4_]^−^, hexafluorophosphate [PF_6_]^−^, thiocyanate [SCN]^−^, and bis(trifluoromethylsulfonyl)imide [Tf_2_N]^−^ by molecular simulation and indicated that the [BMIM][SCN]/IRMOF-1 to be the most favorable site for CO_2_ adsorption. However the use of ILs impregnated on MOF for CO_2_ adsorption study is still limited. In this research, ILs-impregnated AlTp was synthesized for CO_2_ adsorption. The CO_2_ adsorption in these ILs-impregnated AlTp was measured using quartz crystal microbalance (QCM) at the temperature of 298.15 K and pressures up to 5 bar. A novel hybrid model has been offered for correlating CO_2_ isotherm, which resulted in good agreement with the experimental data. The efficacy of the type of anion and cation in the ionic liquid on the CO_2_ adsorption was investigated.

## Experimental section

### Materials

*N*-Methylimidazole (> 99%), 4-Methylpyridine (> 98%), Potassium thiocyanate (> 98%), sodium tetrafluoroborate (> 98%), 1-Chlorobutane (> 99%), and 1-Bromobutane (> 99%) were obtained from Sigma–Aldrich. Ethyl acetate (> 99%), Toluene (> 99%), Ethanol (> 99%) and were obtained from Merck. Aluminum nitrate (Al(NO_3_)_3_·9H_2_O) (> 99%), and Terephthalic acid (> 98%) were purchased from Merck products. CO_2_ gas (> 99%) was used in gas absorption tests.

### Synthesis of components

#### Synthesis of AlTp

AlTp was synthesized using the hydrothermal technique as described in the literature^29^. To synthesize AlTp, 16.71 g (45 mmol) of aluminum nitrate (Al(NO_3_)_3_·9H_2_O), 3.74 g (22.5 mmol) terephthalic acid, and deionized water (50 mL) were put in autoclave at 493 K for about 72 h. The pH of the composition should be in the range of 2–3; otherwise, HNO_3_ is added to achieve the desired pH. The synthesized AlTp with a structural unit of Al(OH)[O_2_C–C_6_H_4_–CO_2_].[HO_2_C_6_H_4_–CO_2_H]x was filtered and washed several times with deionized water to ensure the elimination of unreacted materials and dried in a vacuum for 2 h. The product was calcined for 72 h at 633 K under air for removing terephthalic acid and water molecules trapped inside its cavities.

### Synthesis of the ionic liquids

The ionic liquids, 1-butyl-3-methylimidazolium ionic liquids, ([BMIm][X], and 1-butyl-4-methyl pyridinium ionic liquids, [BMPyr][X] where X = $[Br]^{ - }$, $[Cl]^{ - }$,$[BF_{4} ]^{ - }$, and $[SCN]^{ - }$ were synthesized with a reported method in the literature^30–32^. The procedure to synthesize and purify the ionic liquids was similar to that described previously^33–35^. The water amount of the ionic liquids was evaluated by a Karl-Fischer titrator (720-KSS-Metrohm Herisau, Switzerland). The ^1^H NMR and the spectra of FT-IR of the synthesized ionic liquids are displayed in Figs. S1–S7 (Supporting Information).

### Synthesis of IL-impregnated on MOF

**Figure 1:**
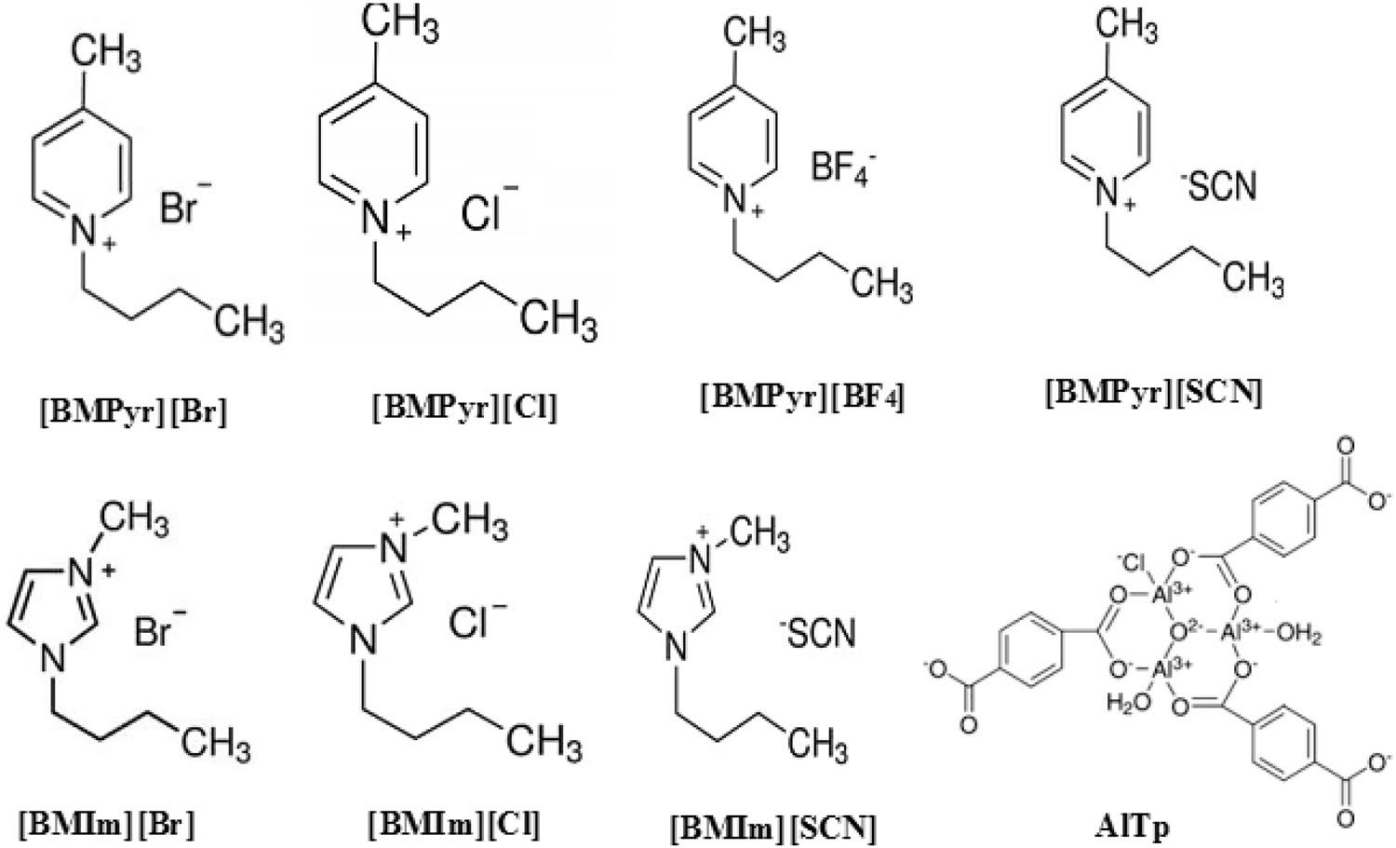
Structures of AlTp and ILs.

AlTp was impregnated by the ILs solution with a ratio of 1:1 wt/vol using a vacuum impregnation method; hence, obtaining 5% ILs on AlTp. The slurry obtained was dried using an oven at 378 K for 24 h. The structures of AlTp and ILs are shown in Fig. 1.

### Characterization of MOF

FTIR spectra were obtained by PerkinElmer (Spectrom RXI). The thermogravimetry analysis (TGA) curve has been determined using a TGA instrument, (TGA-DAT, perkin elmer pyris diamond). A rate of 10 °C/min was used together with nitrogen purging. Furthermore, the BET surface area of the AlTp was also obtained by measuring the nitrogen adsorption at 77 K by a BET (Chem BET-3000) system. The morphology of synthesized MOF was investigated using providing SEM images through LEO 1430VP. X-ray diffraction (XRD) patterns were determined by Philips (X-Pro) in the scan range of 2θ = 5°–70°.

### Gas adsorption apparatus

QCM sensor was applied for gas adsorption measurement. The cell of adsorption entails an 8 MHz AT-cut quartz crystal applied in the electrical oscillator circuit. Adsorption apparatus performance has been mentioned in the prior papers by authors in detail^36–38^. The adsorption capacity of adsorbent, $Q_{e}$ ($mg_{{CO_{2} }} .g_{ILs/AlTp}^{ - 1}$) was computed as follows:

1 $$Q_{e} = \frac{{\Delta F_{S} }}{{\Delta F_{C} }} \times 1000$$

where $\Delta F_{C}$ difference between the coated and the uncoated crystal frequencies. $\Delta F_{S}$ is the difference between the frequencies ILs/AlTp coated crystal under vacuum and the ILs/AlTp coated crystal after CO_2_ adsorption.

### Thermodynamic model

To achieve the isotherm diagrams, the adsorbed gases' amounts and their corresponding equilibrium pressure of adsorption were modeled. The two-parameter models such as Langmuir^39^, Tempkin ^40^, and Freundlich^41^, and three parameters such as Khan ^42^, Redlich-Peterson ^43^, and Sips ^44^ are offered to model the experimental data. Among these isotherm models, the three-parameter Khan model was applied to correlate the data of CO_2_ adsorption on AlTp. In this study, to correlate the experimental data of CO_2_ solubility in IL-impregnated on MOF are used a hybrid law of Henry and Khan Model as below:

2 $$Q= \frac{{p }}{H} + q_{s} \frac{bp}{{(1 + bp)^{n} }}$$

where $p$ is the partial pressure of CO_2_, *H* is the CO_2_ Henry’s law constant, *n* is the dimensionless adsorbent parameter, *q*_*s*_ and *b* are also parameters of the Khan model. The first term in Eq. (2) is Henry law and indicates absorption gas in the confinement IL in the pores of adsorbent. The second term in Eq. (2) is Khan Model indicates adsorption gas in the surface of adsorbent.

## Results and discussion

### Characterization

#### FT-IR spectra

**Figure 2:**
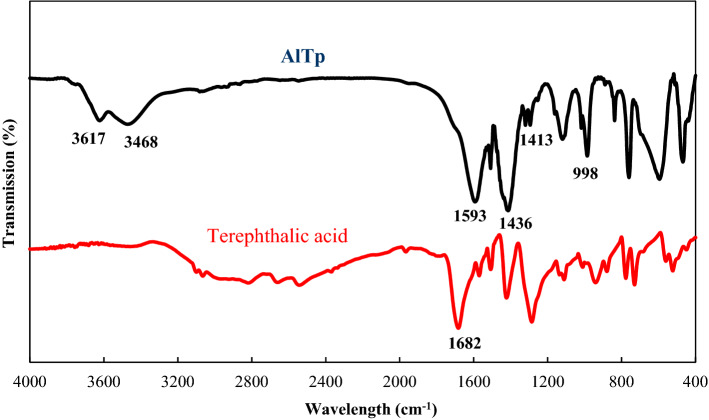
FTIR spectra of the synthesized AlTp.

The FT-IR spectra of the terephthalic acid and AlTp are shown in Fig. 2. As seen in the AlTp spectrum, the broad signals at around 3600–3400 cm^−1^ correspond to the stretching vibration of water molecules poached in the cavities of AlTp. The observed peaks at around 1508 and 1593 cm^−1^ are attributed to C=O asymmetric stretching. These peaks are consistent with the presence of CO_2_ groups that are coordinated to Al. The peaks of the symmetric stretching vibration of –CO_2_ are indicated at 1413 and 1436 cm^−1^ in the AlTp spectrum^45,46^. Moreover, the presence of the Al–O bond (C=O of terephthalate with Al ions) in the AlTp structure was corroborated by the observed signal at 998 cm^−1^. As is presented in Fig. 2 the peak at 1682 cm^−1^ is attributed to the stretching vibration of free carboxylic acids in the terephthalic acid which this peak is eliminated in the spectrum of AlTp. This shows that the terephthalic acids enclosed in AlTp cavities were removed in sample calcinations^47,48^.

### Thermogravimetric analysis

**Figure 3:**
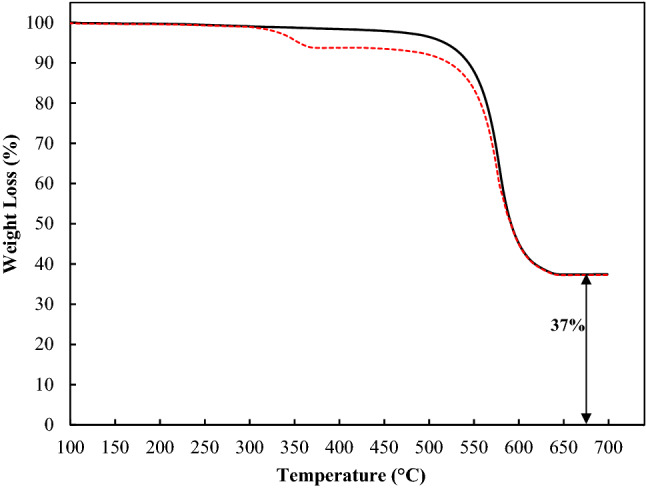
Thermogravimetry analysis (TGA) curve for (dashed line) [BMPyr][Cl]/AlTp and (straight line) AlTp.

Thermogravimetric analysis (TGA) was performed to evaluate the thermal stability of the AlTp and [BMPyr][Cl]/AlTp. The TGA curve for the AlTp and [BMPyr][Cl]/AlTp are shown in Fig. 3. According to Fig. 3, The AlTp structure is stable up to 500 °C, finally collapses with the motion of the bound terephthalic acid. The temperature of 700 °C is related to the amorphous form of Al_2_O_3_ which is in good agreement with reported data^49^. Thermogravimetric (TG) analysis of [BMPyr][Cl]/AlTp shown in Fig. 3 confirmed that the thermal stabilities is stable up to 300 °C. From 300 to 400 °C, it loses about 5% of its weight, which is related to the destruction of the ILs-impregnated, which indicates that 5% of the ionic liquid has been impregnated.

### XRD pattern

**Figure 4:**
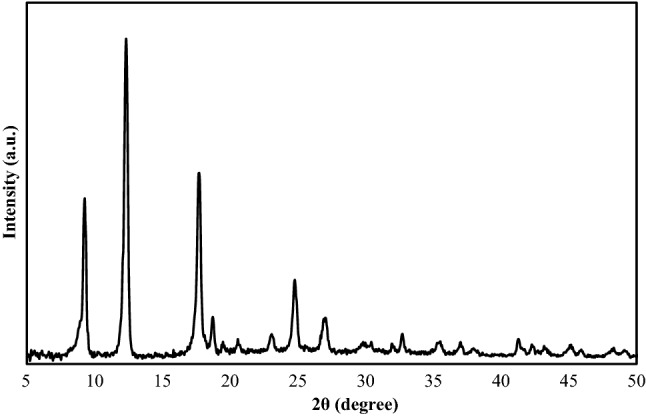
XRD patterns of the synthesized AlTp.

XRD pattern of the AlTp is illustrated in Fig. 4. The crystalline structures of AlTp demonstrate the main diffraction peaks of the AlTp at 2θ = 9.48°, 12.55°, 17.92°, 23.32°, 25.12°, and 27.28°.

### Scanning electron microscopy

**Figure 5:**
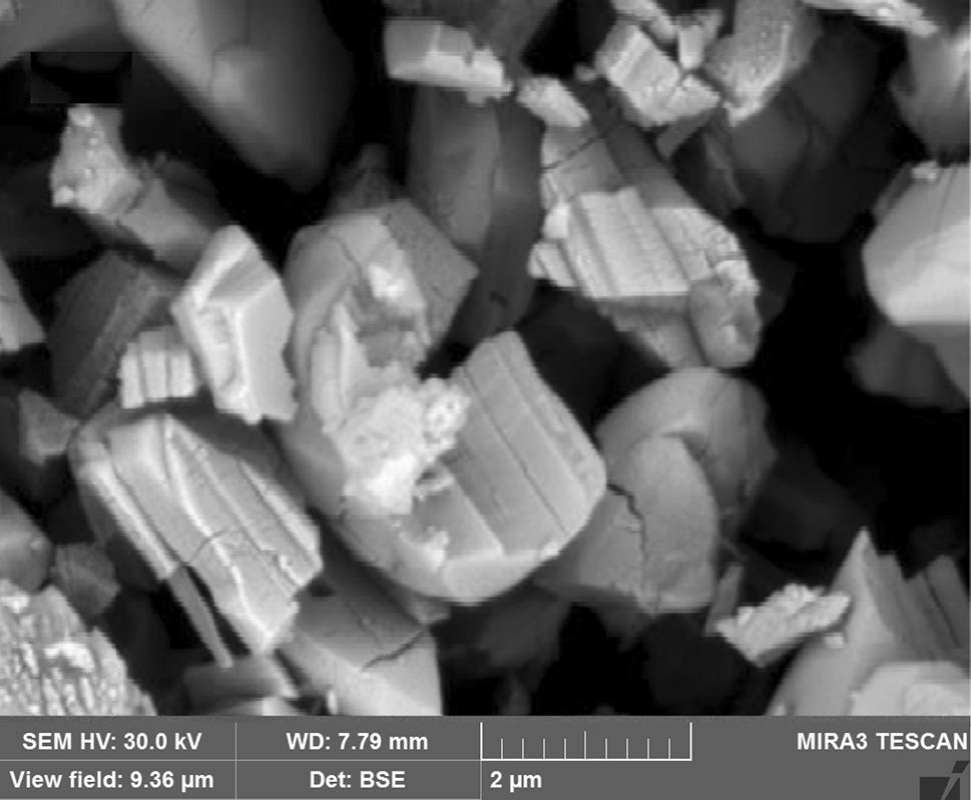
SEM image of the synthesized AlTp.

Scanning Electron Microscopy (SEM) was used to determine crystal morphology and size of the products. The SEM image of the AlTp is shown in Fig. 5. As seen in the SEM image, the AlTp species is seen as layered cubes with small pores.

### BET analysis

**Figure 6:**
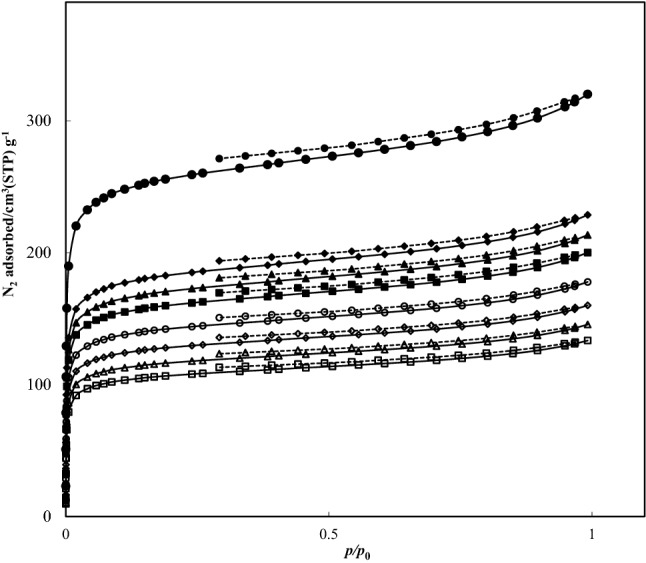
Nitrogen desorption–adsorption BET isotherms at 77 K: in (filled circle) AlTp; (filled diamond) [BMIm][Cl]/AlTp; (filled triangle)[BMIm][Br]/AlTp; (filled square)[BMIm][SCN]/AlTp; (open circle)[BMPyr][Cl]/AlTp; (open diamond)[BMPyr][Br]/AlTp; (open triangle) [BMPyr][SCN]/AlTp; and (open square)[BMPyr][BF_4_]/AlTp; (straight line) N_2_ adsorption and (dotted line) N_2_ desorption.

Nitrogen adsorption isotherms displayed for AlTp and ILs/AlTp at 77 K in Fig. 6. The results obtained from BET analysis were also presented in Table 1. According to the results plotted in Fig. 5, the amount of adsorbed N_2_ by ILs/AlTp was significantly less than that AlTp. Moreover, the specific surface area (A_BET_) and total pore volume (V_P_) of ILs/AlTp were lower than those of AlTp. The micropore volume and surface area of MOF is comparable with values reported in the literature^50^. The textural properties indicated that the impregnation procedure decreased the values of micropore volume, specific surface area and total pore volume relative to the original AlTp sample. This behavior reveals that ILs molecules were incorporated in the pores of AlTp because of the impregnation process.

**Table 1:** Textural characteristics of the samples.

ILs/AlTp	A_BET_ (m^2^·g^−1^)	V_P_ (cm^3^·g^−1^)
AlTp	985.72	0.49
[BMIm][Cl]/AlTp	780.45	0.45
[BMIm][Br]/AlTp	725.65	0.44
[BMIm][SCN]/AlTp	664.56	0.37
[BMPyr][Cl]/AlTp	578.35	0.35
[BMPyr][Br]/AlTp	534.80	0.33
[BMPyr][SCN]/AlTp	496.35	0.29
[BMPyr][BF_4_]/AlTp	420.38	0.27

### Adsorption isotherms

**Figure 7:**
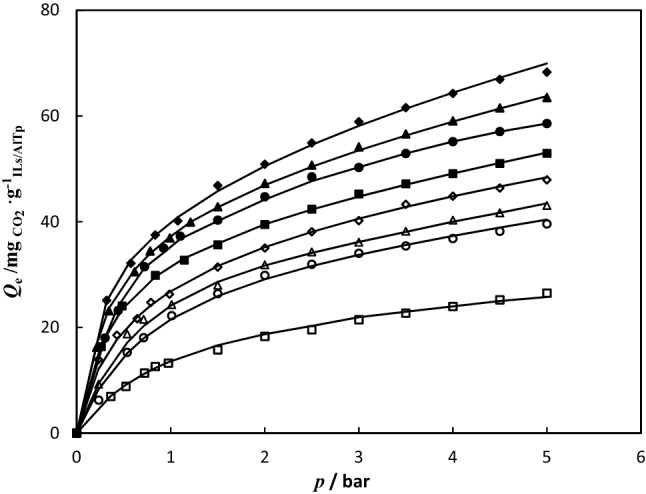
The CO_2_ adsorption in (filled diamond) [BMPyr][Cl]/AlTp; (filled triangle) [BMIm][Cl]/AlTp; (filled circle) [BMPyr][Br]/AlTp; (filled square) [BMIm][Br]/AlTp; (open diamond) [BMPyr][SCN]/AlTp; (open triangle) [BMIm][SCN]/AlTp; (open circle)[BMPyr][BF_4_]/AlTp at temperature 298.15 K; (straight line) Fitting results by Eq. (2).

**Figure 8:**
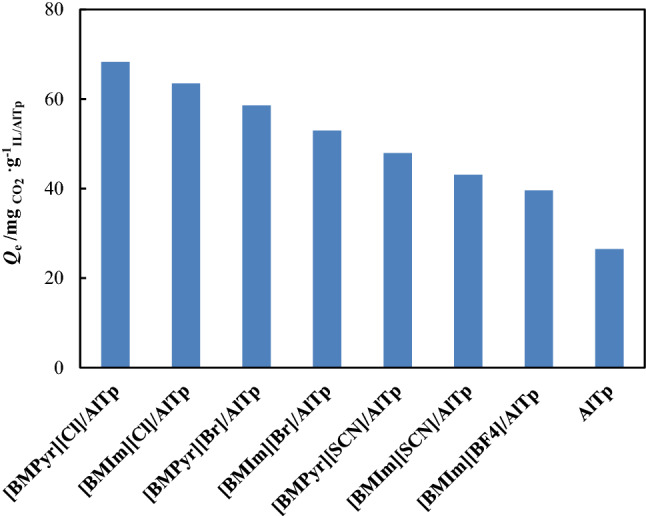
Graphical comparison of CO_2_ adsorption capacity in the different IL/AlTp systems at temperature 298.15 K and pressure 5 bar.

CO_2_ adsorption in ILs-impregnated AlTp MOF was measured at a temperature of 298.15 K and pressure of up to 5 bar. The CO_2_ adsorption data are tabulated in Table 2. The experimental data of CO_2_ solubility in IL-impregnated on AlTp is correlated by the hybrid model (Eq. 2). The parameters of the Khan Model, Henry’s law constant, and absolute average relative deviation (*AARD*) for experimental adsorption data for CO_2_ on the synthesized ILs/AlTp are reported in Table 3. The absolute average relative deviation is lower than 0.02, which implies to suitable capability of the proposed model. According to the data, the modification of AlTp MOF with ILs increases the CO_2_ adsorption capacity. At pressure of 5 bar, AlTp has a CO_2_ adsorption capacity of 26.46 $mg_{{CO_{2} }} .g_{AlTp}^{ - 1}$, while ILs-impregnated AlTp [BMPyr][Cl]/AlTp, [BMIm][Cl]/AlTp, [BMPyr][Br]/AlTp, [BMIm][Br]/AlTp, [BMPyr][SCN]/AlTp, [BMIm][SCN]/AlTp, and [BMPyr][BF_4_]/AlTp had adsorption capacities of ca. 68.27, 63.48, 58.56, 52.93, 47.91, 43.05 and 39.58 $mg_{{CO_{2} }} .g_{ILs/AlTp}^{ - 1}$, respectively. The CO_2_ adsorption isotherm in ILs-impregnated AlTp was illustrated at a temperature of 298.15 K and pressure of up to 5 bar in Fig. 7. In AlTp main interaction between CO_2_ and AlTp arises from carboxylate oxygen atoms and unsaturated metal centers (UMCs), can increase preferential interactions between the frameworks and CO_2_. In the MOF impregnated with ILs, at low pressure gas adsorption take placed on the immobilized ILs on surface of MOF; whereas at high pressure gas adsorption take placed on the confinement ILs on pores of MOF. According Fig. 7, the CO_2_ adsorption in [BMPyr][SCN]/AlTp, [BMPyr][Cl]/AlTp, and [BMPyr][Br]/AlTp is more than the CO_2_ adsorption in [BMIm][SCN]/AlTp, [BMIm][Cl]/AlTp, and [BMIm][Br]/AlTp. This behavior may be attributed to the stabilization of charge distribution in cation of ILs. The cation in pyridinium-based ionic liquids has a hexagonal ring whereas the cation in imidazolium-based ionic liquids has a pentagonal ring; therefore stabilization of charge distribution in cation of pyridinium-based ionic liquids is done better than that the cation in imidazolium-based ionic liquids. This behavior causes weak interactions between cation and anion in pyridinium-based ionic liquids and the consequence increases the interactions between CO_2_ and IL; therefore this leads to an increment in the CO_2_ solubility. Also, the comparison of CO_2_ adsorption in ILs/AlTp with different anion of ILs**,** viz. chloride ([Cl]^−^), bromide ([Br]^−^), thiocyanate ([SCN]^−^), and tetrafluoroborate [BF_4_]^−^ in Fig. 8 depicts CO_2_ adsorption in ILs/AlTp was increased with different anion of ILs in the order as: [BF_4_]^−^ < [SCN]^−^ < [Br]^−^ < [Cl]^−^. This trend implies that anion acts a main role in the CO_2_ adsorption in ILs/AlTp. In the MOF impregnated with ILs, gas adsorption arises from two factor; first factor is ILs which confinement in pores and second factor is ILs which immobilized in the surface of pores. In the ILs with large size anion possibility of confinement in pores reduced. Therefore in ILs with halide anion gas adsorption take placed due to confinement and immobilized ILs in pores but in the ILs with [BF_4_]^−^ anion gas adsorption take placed only due to immobilized ILs. Therefore adsorption capacity in MOF impregnated with halide anion is higher than [BF_4_]^−^, nevertheless CO_2_ adsorption in pure [BF_4_]^−^ ILs higher than halides.

**Table 2:** CO_2_ adsorption capacity $Q_{e}$ ($mg_{{CO_{2} }} .g_{ILs/AlTp}^{ - 1}$) of ILs/AlTp at 298.15 K and pressures up to 5 bar.

*T* = 298.15 K		*T* = 298.15 K		*T* = 298.15 K		*T* = 298.15 K	
*p* (bar)	$Q_{e}$ ($mg_{{CO_{2} }} .g_{ILs/AlTp}^{ - 1}$)	*p* (bar)	$Q_{e}$ ($mg_{{CO_{2} }} .g_{ILs/AlTp}^{ - 1}$)	*p* (bar)	$Q_{e}$ ($mg_{{CO_{2} }} .g_{ILs/AlTp}^{ - 1}$)	*p* (bar)	$Q_{e}$ ($mg_{{CO_{2} }} .g_{ILs/AlTp}^{ - 1}$)
AlTp		[BMPyr][Cl]/AlTp		[BMIm][Cl]/AlTp		[BMPyr][Br]/AlTp	
0.362	6.931	0.318	25.112	0.212	16.240	0.302	18.000
0.524	8.822	0.576	32.129	0.343	23.130	0.441	23.189
0.722	11.342	0.836	37.483	0.615	30.512	0.722	31.486
0.836	12.602	1.076	40.161	0.781	34.449	0.924	35.005
0.975	13.233	1.500	46.854	0.990	36.925	1.102	37.264
1.500	15.753	2.000	50.870	1.210	39.894	1.500	40.302
2.000	18.273	2.500	54.886	1.500	42.815	2.000	44.710
2.500	19.534	3.000	58.902	2.000	47.244	2.500	48.489
3.000	21.424	3.500	61.580	2.500	50.689	3.000	50.256
3.500	22.684	4.000	64.257	3.000	54.134	3.500	52.897
4.000	23.945	4.500	66.934	3.500	56.594	4.000	55.156
4.500	25.205	5.000	68.273	4.000	59.055	4.500	57.048
5.000	26.465			4.500	61.516	5.000	58.564
				5.000	63.484		

[BMIm][Br]/AlTp		[BMPyr][SCN]/AlTp		[BMIm][SCN]/AlTp		[BMPyr][BF_4_]/AlTp	
0.261	16.362	0.231	7.728	0.234	4.861	0.234	4.861
0.483	24.062	0.428	12.365	0.537	10.417	0.537	8.333
0.836	29.836	0.643	15.456	0.713	13.194	0.713	10.417
1.144	32.724	0.789	18.547	1.010	15.972	1.010	12.500
1.500	35.611	0.986	20.093	1.500	19.444	1.500	15.972
2.000	39.461	1.500	24.730	2.000	22.917	2.000	18.056
2.500	42.348	2.000	27.821	2.500	25.694	2.500	20.139
3.000	45.236	2.500	30.912	3.000	27.778	3.000	22.222
3.500	47.161	3.000	32.458	3.500	29.861	3.500	23.611
4.000	49.086	3.500	35.549	4.000	31.944	4.000	25.000
4.500	51.011	4.000	37.094	4.500	33.333	4.500	26.389
5.000	52.936	4.500	38.640	5.000	34.722	5.000	27.778
		5.000	40.185				

Standard uncertainties are u ($Q_{e}$) = 0.001, u(*T*) = 0.05 K, and u (*p*) = 0.001.

**Table 3:** Henry’s law constant (*H),* $q_{s}$ and *b* are also parameters of the Khan model, the dimensionless adsorbent parameter (*n*), the correlation coefficient (*R*^2^) and absolute average relative deviation (*AARD*) for CO_2_ adsorption ILs/AlTp at 298.15 K.

ILs/AlTp	*T* (K^a^)	*H* (bar)	$q_{s}$ ($mg_{{CO_{2} }} .g_{ILs/AlTp}^{ - 1}$)	*b* (${\text{bar}}^{ - 1}$)	*n*	*R*^*2*^	^b^*AARD*%
AlTp	298.15	–	26.553	0.966	0.938	0.99845	1.62
[BMPyr][Cl]/AlTp	298.15	0.434	21.138	11.789	0.741	0.99858	1.90
[BMIm][Cl]/AlTp	298.15	0.449	32.039	3.582	0.806	0.99924	1.05
[BMPyr][Br]/AlTp	298.15	0.482	26.716	6.039	0.81	0.99938	0.99
[BMIm][Br]/AlTp	298.15	0.537	33.278	4.476	0.902	0.99961	0.78
[BMPyr][SCN]/AlTp	298.15	0.561	24.121	3.626	0.847	0.99897	1.38
[BMIm][SCN]/AlTp	298.15	0.566	28.119	0.659	0.915	0.99909	1.27
[BMPyr][BF_4_]/AlTp	298.15	0.626	39.282	1.074	0.875	0.99925	1.09

^a^Standard uncertainty is u(*T*) = 0.05 K.

^b^$$AARD\% = \frac{100}{n}\sum {\left| {\frac{{Q_{{CO_{2} }}^{cal} - Q_{{CO_{2} }}^{\exp } }}{{Q_{{CO_{2} }}^{\exp } }}} \right|}$$.

### Regeneration efficiency of IL/AlTp

**Figure 9:**
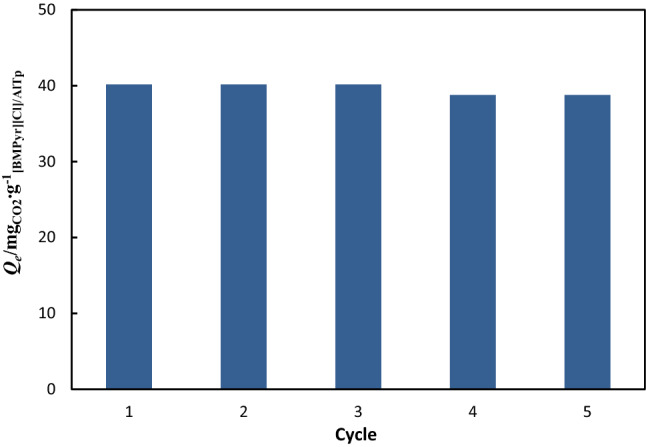
The CO_2_ absorption capacity of [BMPyr][Cl]/AlTp at *p* = 1 bar and *T* = 298.15 K in five regeneration cycles.

Regeneration efficiency was calculated to estimate the desorption performance of the CO_2_ adsorbent in ILs/AlTp. To evaluate reuse capacity, five cycles of CO_2_ adsorption/desorption test [BMPyr][Cl]/AlTp are illustrated in Fig. 9. For the regeneration test, CO_2_ adsorption has tested at 298.15 K and 1 bar and, vacuum desorption at 298.15 K and 90 min for CO_2_ elimination. The value of absorption/desorption shows that CO_2_ is entirely eliminated in 90 min and the amount of adsorption is constant in five cycles. The CO_2_ adsorption capacity reduction in the regenerated [BMPyr][Cl]/AlTp compared to the fresh sample was estimated at about 1% in 5 cycles. This trend confirms that the ILs/AlTp is stable and regenerable under the practical condition of regeneration. The values of CO_2_ adsorption [BMPyr][Cl]/AlTp are calculated 40.161, 40.161, 39.547, 38.770, and 38.770 in five consecutive cycles of adsorption/desorption and regeneration performance of [BMPyr][Cl]/AlTp is 96.53% after five consecutive cycles adsorption/desorption.

## Conclusions

Aluminum terephthalate metal–organic framework (AlTp) impregnated of 1-butyl-4-methyl pyridinium and 1-butyl-3-methylimidazolium–based ionic liquids with different anions, tetrafluoroborate ([BF_4_]^-^), thiocyanate ([SCN]^-^), chloride ([Cl]^-^), and bromide ([Br]^-^) were synthesized and characterized by SEM, FT-IR, XRD, TGA and BET techniques. CO_2_ adsorption in AlTp impregnated with ILs was studied at 298.15 K and pressures up to 5 bar using QCM. The hybrid law of Henry and Khan Model has been applied to evaluate CO_2_ absorption in the AlTp impregnated of ILs. Comparison of CO_2_ adsorption in ILs/ AlTp with different anion ([Cl]^−^, [Br]^−^, [SCN]^−^, [BF_4_]^−^) reveals anion play importance role in CO_2_ adsorption. The results show that [BMPyr][Cl]/AlTp had the highest CO_2_ adsorption capacity among the studied systems. Also, after several adsorption/desorption test regeneration efficiency slightly reduced; therefore it can be concluded that these system were reusable and logical for gas separation applications.

## Supplementary Information

Supplementary Figures.

## Data Availability

The datasets used and/or analyzed during the current study are available from the corresponding author on reasonable request.
