# Supplementary material for: Improving performance of mesoporous MOF AlTp impregnated with ionic liquids for CO2 adsorption
Source: Sci Rep. 2023 Feb 24;13:3227. doi: 10.1038/s41598-023-30267-x (PMC9958030; doi:10.1038/s41598-023-30267-x)
Supplement: Supplementary file 1 — Supplementary Figures. [file 41598_2023_30267_MOESM1_ESM.pdf]

Supporting Information

Improving performance of mesoporous MOF AlTp impregnated with ionic liquids for CO_2 adsorption

Narmin Noorani, Abbas Mehrdad*

Department of Physical Chemistry, Faculty of Chemistry, University of Tabriz, Tabriz, Iran

Corresponding Author, E–mail address: a_mehrdad@tabrizu.ac.ir

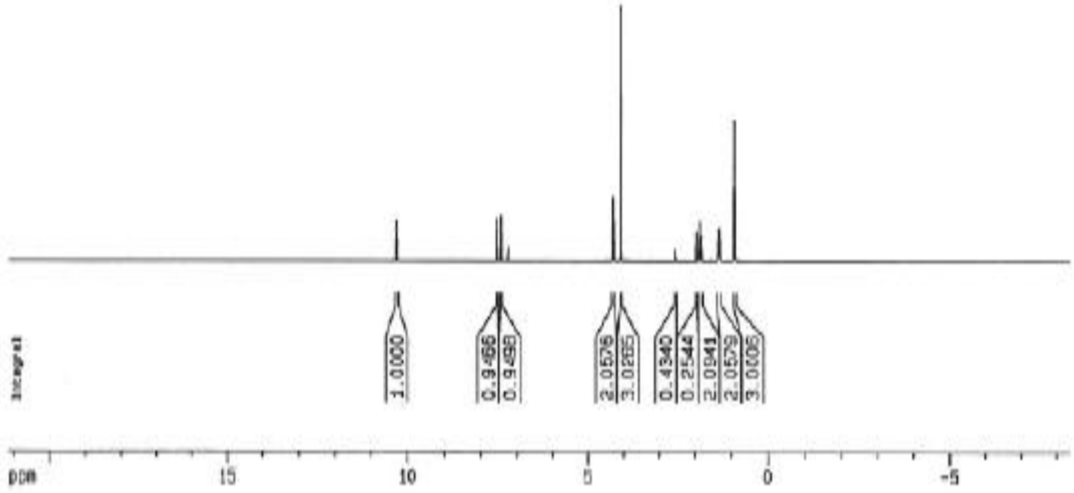

Figure S1. ^{1}HNMR data of 1-butyl-3-methyl imidazolium bromide: ^{1}HNMR (400MHz: $CDCl_3$; δ/ppm relative to TMS):0.91 (3H, t, but-CH_3), 1.34 (2H, m, CH_2), 1.86 (2H, m, CH_2), 4.08 (3H, s, NCH_3), 4.29 (2H, t, NCH_2), 7.46(1H, s, NCH),7.58 (1H, s, NCH), 10.30 (1H, s, NCHN).

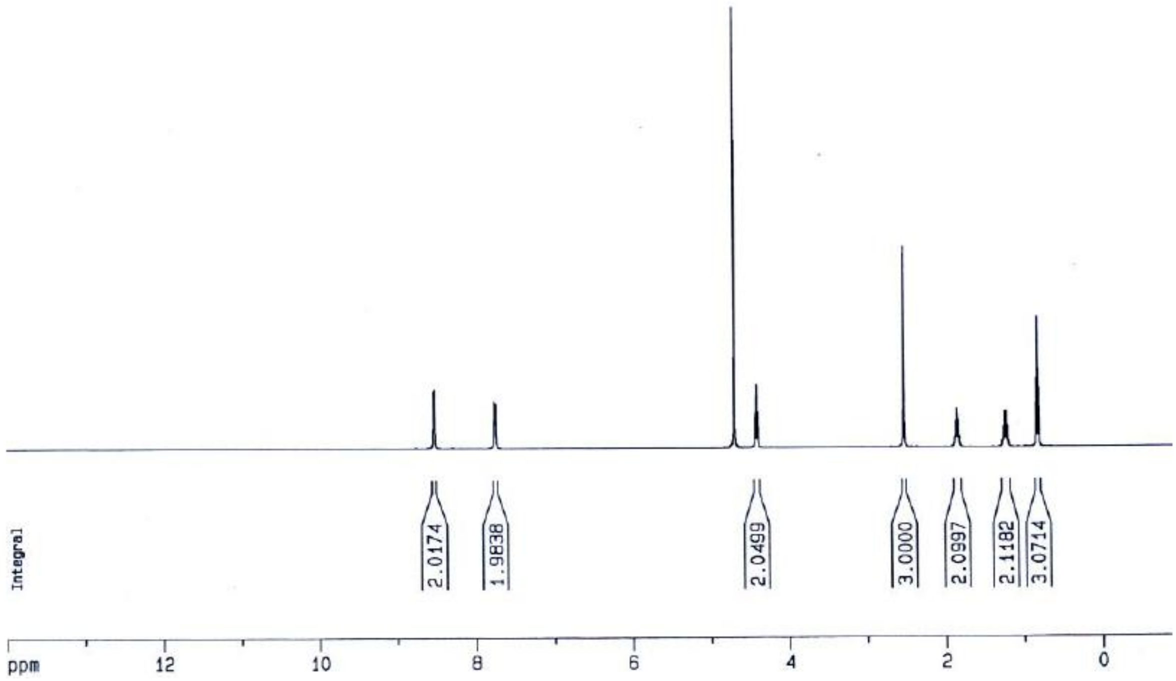

Figure S2. ^{1}H-NMR data of 1-butyl-4-methylpyridinium bromide: ^{1}H-NMR (400MHz: D_2O; δ/ppm relative to TMS): 0.81 (3H, but-CH_3), 1.24 (2H, CH_2), 1.85 (2H, CH_2), 2.53 (3H, C-CH_3), 4.42 (2H, N-CH_2), 7.75(2H, CH), 8.53 (2H, N-CH).

Additionally, the spectrum shows a signal at 4.7 ppm (D_2O, HDO).

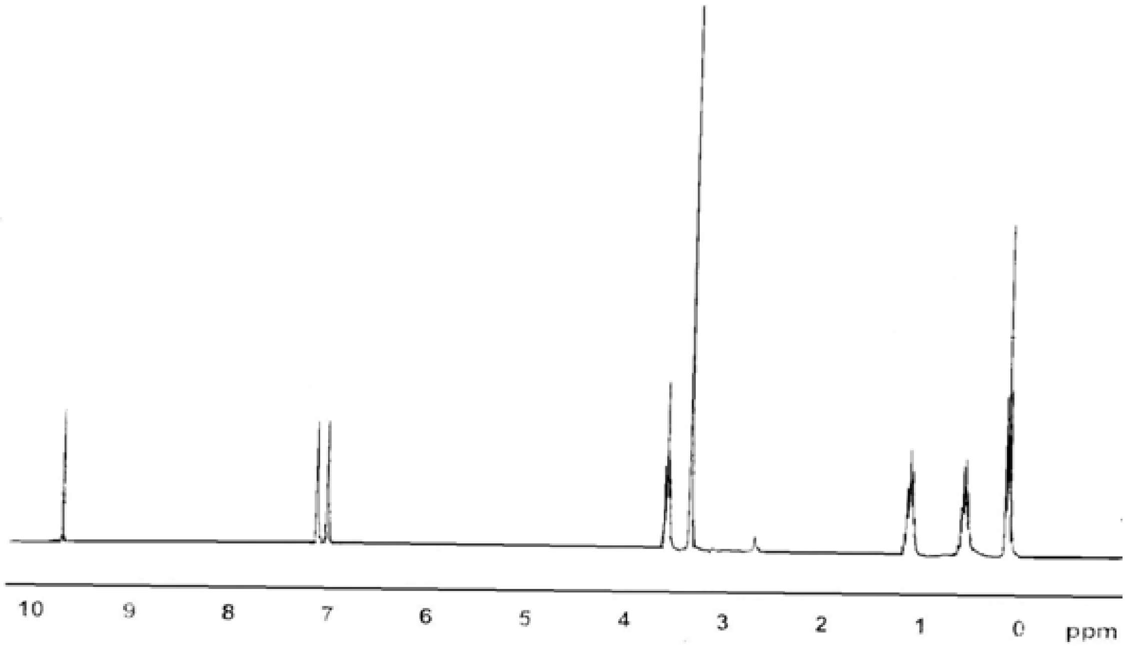

Figure S3. ^{1}HNMR data of 1-butyl-3-methyl imidazolium chloride: ^{1}HNMR (400MHz: $CDCl_3$; δ/ppm relative to TMS): 0.16 (3H, t, but-CH_3), 0.71 (2H, m, CH_2), 1.13 (2H, m, CH_2), 3.36 (3H, s, NCH_3), 3.59 (2H, t, NCH_2), 7.13(1H, s, NCH),7.02 (1H, s, NCH), 9.69 (1H, s, NCHN).

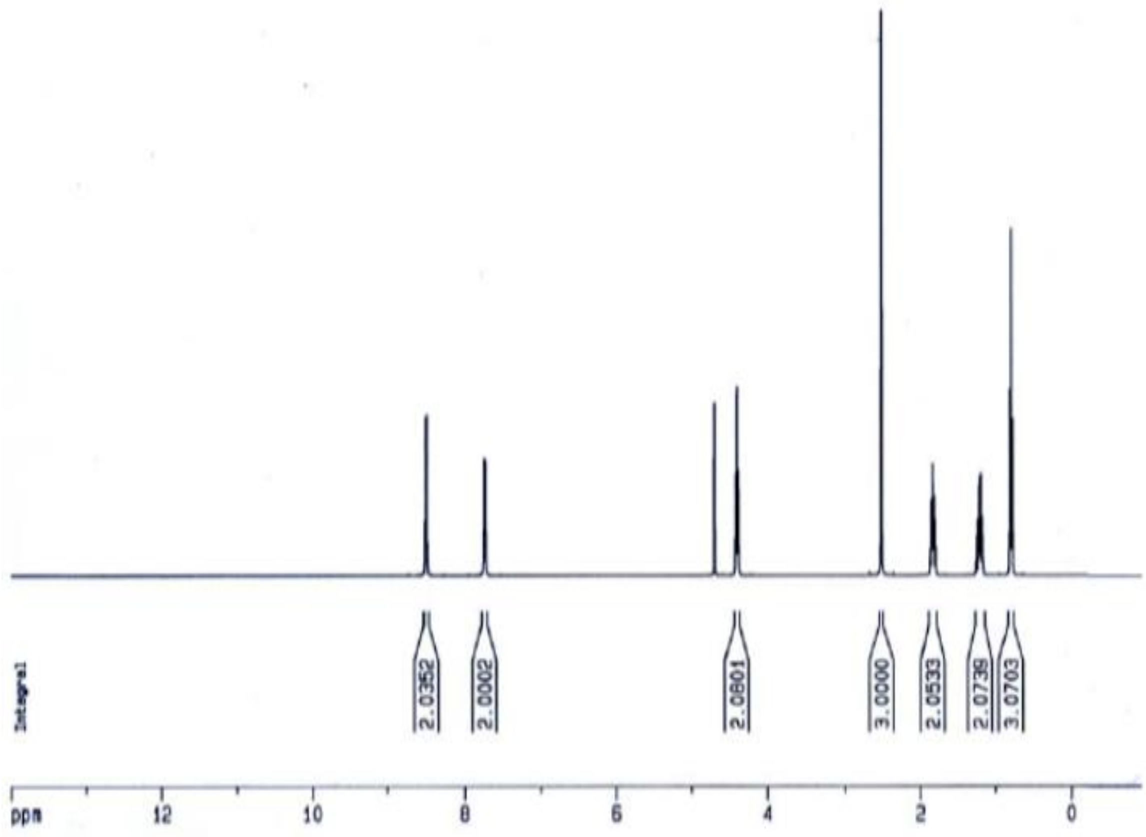

Figure S 4. ^{1}HNMR data of 1-butyl-4-methylpyridinium chloride: ^{1}HNMR (400MHz: D_2O; δ/ ppm relative to TMS): 0.79 (3H, but-CH_3), 1.22 (2H, CH_2), 1.82 (2H, CH_2), 251 (3H, C-CH_3), 4.40 (2H, N-CH_2), 7.73 (2H, CH), 8.51 (2H, N-CH).

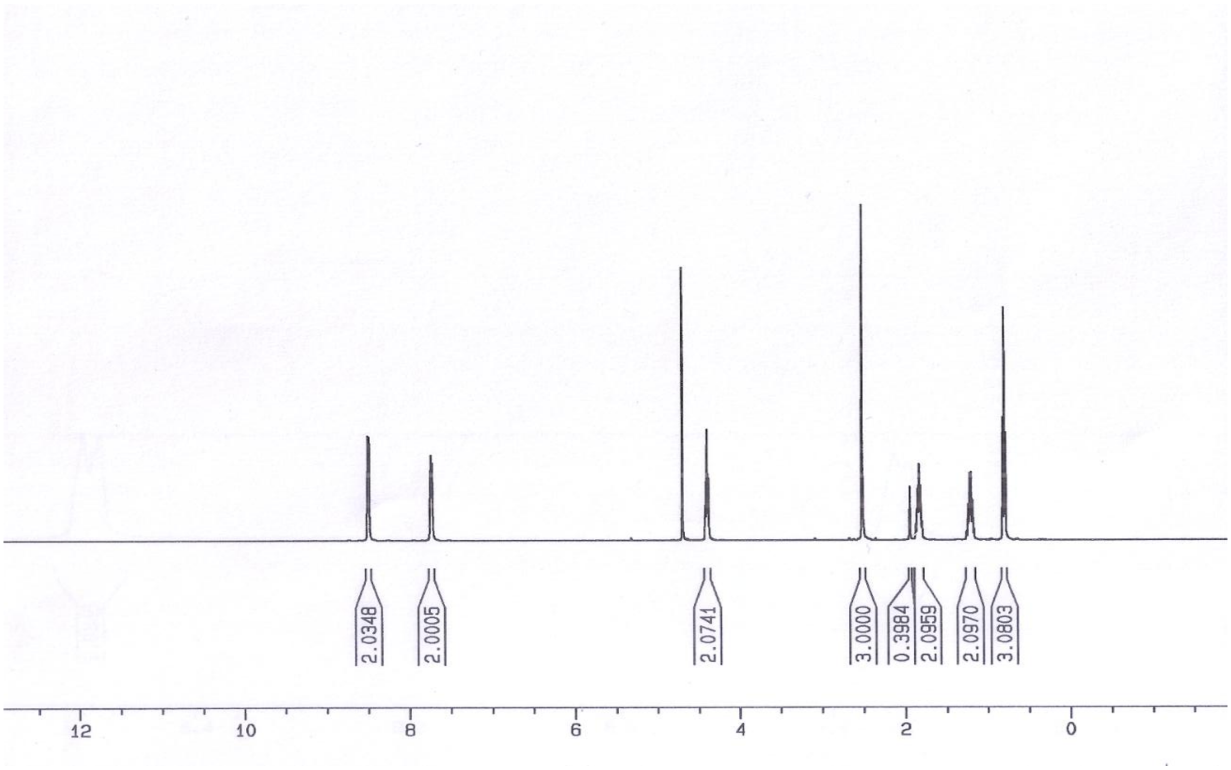

Figure **S5.** ^{1}H NMR data of 1–butyl–4–methylpyridinium tetra flouro bourate: $^{1}HNMR$ (400MHz: D_2O; δ/ ppm relative to TMS): 0.80 (3H, but-CH_3), 1.216 (2H, CH_2), 1.82 (2H, CH_2), 2.51 (3H, C-CH_3), 4.70 (2H, N-CH_2), 7.73 (2H, CH), 8.49 (2H, N-CH).

Additionally, the spectrum shows a signal at 4.7 ppm (D_2O, HDO).

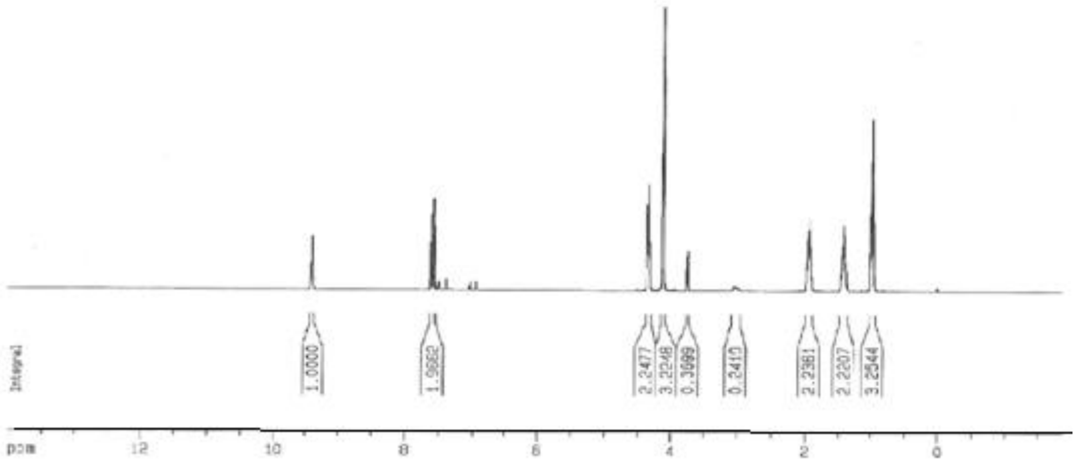

Figure S6. ^{1}HNMR data of 1-butyl-3-methyl imidazolium thiocyanate: ^{1}HNMR (400MHz: $CDCl_3$; δ/ppm relative to TMS): 0.97 (3H, t, but-CH_3), 1.42 (2H, m, CH_2), 1.95 (2H, m, CH_2), 3.73 (3H, s, NCH_3), 4.31 (2H, t, NCH_2), 7.01(1H, s, NCH),7.61 (1H, s, NCH), 9.38 (1H, s, NCHN).

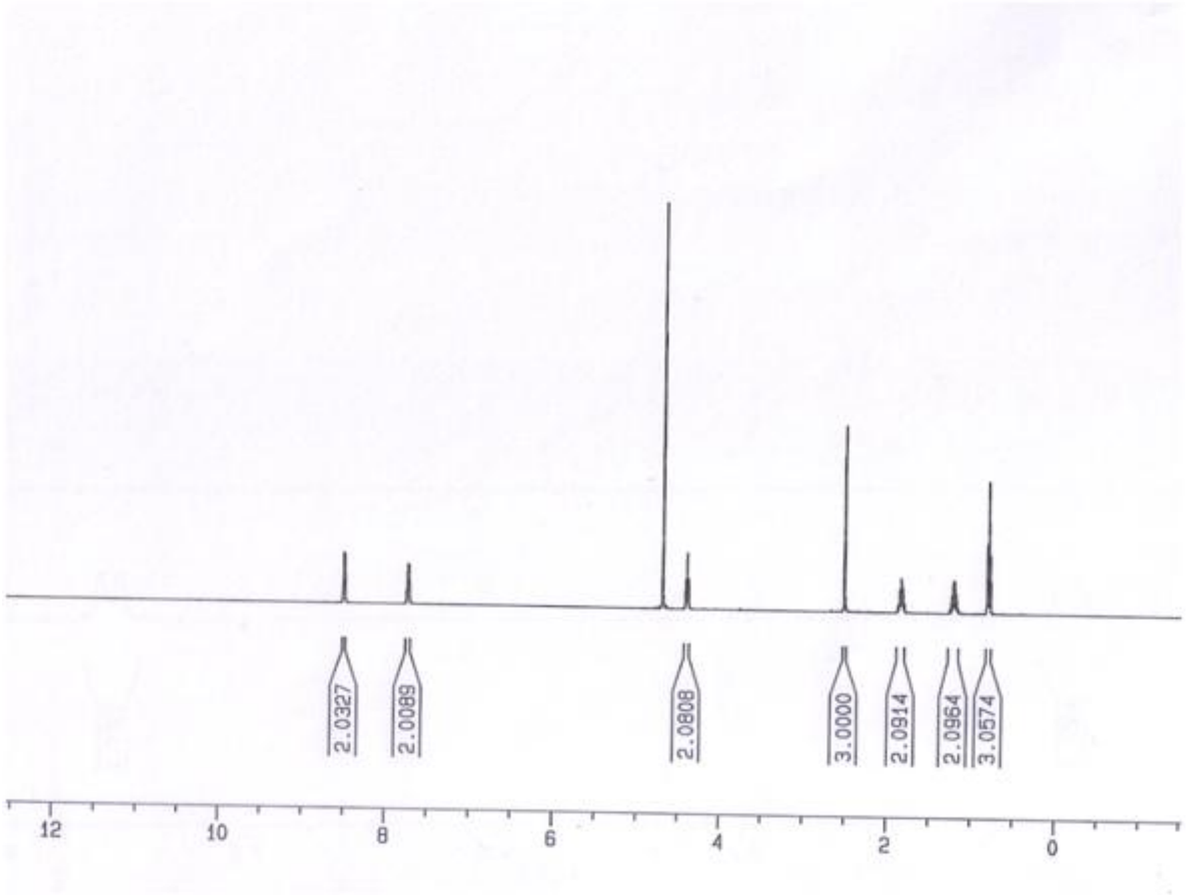

Figure S7. ^{1}HNMR data of 1–butyl–4–methylpyridinium thiocyanate: ^{1}HNMR (400MHz: D_2O; δ/ ppm relative to TMS): 0.80 (3H, but-CH_3), 1.219 (2H, CH_2), 1.84 (2H, CH_2), 2.51 (3H, C-CH_3), 4.70 (2H, N-CH_2), 7.73 (2H, CH), 8.51 (2H, N-CH).
